# Ukrainian Consensus on Diagnosis and Management of Vitamin D Deficiency in Adults

**DOI:** 10.3390/nu16020270

**Published:** 2024-01-16

**Authors:** Nataliia Grygorieva, Mykola Tronko, Volodymir Kovalenko, Serhiy Komisarenko, Tetiana Tatarchuk, Ninel Dedukh, Mykola Veliky, Serhiy Strafun, Yulia Komisarenko, Andrii Kalashnikov, Valeria Orlenko, Volodymyr Pankiv, Oleg Shvets, Inna Gogunska, Svitlana Regeda

**Affiliations:** 1 D.F. Chebotarev Institute of Gerontology, The National Academy of Medical Sciences of Ukraine, 04114 Kyiv, Ukraine; 2 V.P. Komisarenko Institute of Endocrinology and Metabolism, The National Academy of Medical Sciences of Ukraine, 04114 Kyiv, Ukraine; 3 National Scientific Center «The M.D. Strazhesko Institute of Cardiology», Clinical and Regenerative Medicine, The National Academy of Medical Sciences of Ukraine, 03151 Kyiv, Ukraine; 4 Palladin Institute of Biochemistry, The National Academy of Sciences of Ukraine, 02000 Kyiv, Ukraine; 5 Institute of Pediatrics, Obstetrics and Gynecology Named after Academician O.M. Lukyanova, The National Academy of Medical Sciences of Ukraine, 04050 Kyiv, Ukraine; 6 Institute of Traumatology and Orthopedics, The National Academy of Medical Sciences of Ukraine, 01601 Kyiv, Ukraine; 7 Department of Endocrinology, O.O. Bogomolets National Medical University, 01601 Kyiv, Ukraine; julia.komissarenko@gmail.com; 8 Ukrainian Scientific and Practical Centre for Endocrine Surgery, Transplantation of Endocrine Organs and Tissues, Health Ministry of Ukraine, 01021 Kyiv, Ukraine; 9 Department of Public Health and Nutrition, National University of Life and Environmental Sciences of Ukraine, 03041 Kyiv, Ukraine; hyppocrat@gmail.com; 10 O.S. Kolomiychenko Institute of Otolaryngology, The National Academy of Medical Sciences of Ukraine, 03057 Kyiv, Ukraine; innagogunskaya@gmail.com; 11 Center of Innovative Medical Technologies, The National Academy of Sciences of Ukraine, 04053 Kyiv, Ukraine; regedas@gmail.com

**Keywords:** Consensus, deficiency, prevention, recommendations, screening, supplementation, treatment, vitamin D, 25-hydroxyvitamin D

## Abstract

Vitamin D deficiency (VDD) is a global problem, however, there were no Ukrainian guidelines devoted to its screening, prevention, and treatment, which became the reason for the Consensus creation. This article aimed to present the Consensus of Ukrainian experts devoted to VDD management. Following the creation of the multidisciplinary Consensus group, consent on the formation process, drafting and fine-tuning of key recommendations, and two rounds of voting, 14 final recommendations were successfully voted upon. Despite a recent decrease in VDD prevalence in Ukraine, we recommend raising awareness regarding VDD’s importance and improving the strategies for its decline. We recommend screening the serum 25-hydroxyvitamin D (25(OH)D) level in risk groups while maintaining a target concentration of 75–125 nmol/L (30–50 ng/mL). We recommend prophylactic cholecalciferol supplementation (800–2000 IU/d for youthful healthy subjects, and 3000–5000 IU/d for subjects from the risk groups). For a VDD treatment, we recommend a short-term administration of increased doses of cholecalciferol (4000–10,000 IU/d) with 25(OH)D levels monitored after 4–12 weeks of treatment, followed by the use of maintenance doses. Additionally, we recommend assessing serum 25(OH)D levels before antiosteoporotic treatment and providing vitamin D and calcium supplementation throughout the full course of the antiosteoporotic therapy.

## 1. Introduction

Vitamin D is a class of fat-soluble biologically active secosteroids, containing more than six vitamers and fifty metabolites, whose role in human health continues to be actively studied [1,2,3,4,5]. It enters the human body through synthesis in the skin under sun exposure and with food and supplements. In the liver, vitamin D metabolizes to 25(OH)D, and then in kidneys (in the majority) and other organs and tissues, biologically active vitamin D metabolite (1,25-dihydroxyvitamin D, 1α,25(OH)_2_D) is created from vitamin D vitamers through 25-hydroxyvitamin D (25(OH)D), which contains 25(OH)D_2_ and 25(OH)D_3_ [3,5,6,7].

Vitamin D has a wide range of skeletal and extra-skeletal effects [8,9] and it is involved in many biological processes through genomic and non-genomic mechanisms [10,11,12]. In particular, vitamin D supports calcium–phosphorus homeostasis, cell proliferation and differentiation in various organs and systems, bone growth, and mineralization [2,8,9]. In almost all organs and tissues of a human, the vitamin D receptor (VDR) has been detected in the nuclei and on cell membranes [13,14], and cells also contain CYP27B1 (Cytochrome P450 Family 27 Subfamily B Member 1) and CYP24A1 (Cytochrome P450 Family 24 Subfamily A Member 1), as well as coactivator/repressor proteins that regulate the transcription of these proteins. This indicates that cells that include these proteins are able to metabolize and synthesize the active form of 1α,25(OH)_2_D_3_.

According to the current view, vitamin D deficiency (VDD) is a clinical syndrome in children and adults caused by low serum 25(OH)D levels [15]. According to the International Classification of Diseases (ICD-10), VDD is classified in the heading E55 (VDD. Excluded: consequences of rickets (E64.3), osteoporosis (M80–M81), and adult osteomalacia (M83)) and E55.9 (VDD, unspecified. Vitamin D Avitaminosis).

Nowadays, numerous epidemiological studies demonstrate a high prevalence of VDD in the world [16,17,18,19,20]. The global VDD rate is about 37% [2], but its frequency depends on the season, age, and sex of the examined, country of residence, genetics, as well as presence and type of comorbidities. The highest VDD prevalence was noted in Iran and Jordan (up to 90% of the population), in European countries up to 40%, consisting of about 20% in Northern Europe and 30–60% in Western, Eastern and Southern Europe [16]. In African countries, VDD accounts for 34%. The lowest rates are recorded in the Seychelles (up to 7%) and the United States (18%) [18].

Current international [15,18,21,22] guides confirmed the great relevance of the VDD problem in the world; however, there were no Ukrainian recommendations for the management of VDD, which became the reason for the Consensus creation.

## 2. Methodology of Consensus Development

The Consensus creation was performed by the multidisciplinary expert group consisting of 15 leading Ukrainian scientists who have extensive experience in studying vitamin D and related topics. The 1st (N.G.), 6th (N.D.), and 11th (V.O.) authors of this article were members of the Task Force group who coordinated the experts’ work. Based on careful analysis of the current literature with a high level of evidence, the Task Force members formulated 14 recommendations regarding VDD epidemiology, its screening, prevention, therapy, and monitoring. Following the agreement on the formation process of the Consensus between experts, the Delphi method, which is widely used for guideline creation [23,24], was chosen. The voting was performed on the SurveyMonkey^®^ (https://www.surveymonkey.com, San Mateo, CA, USA) platform using 9-point grading of agreement with the recommendations (1—strongly disagree, 3—disagree, 5—neutral, 7—agree, and 9—strongly agree). Before voting, the experts agreed that Consensus would be achieved when more than 75% of the experts were in agreement with a recommendation on a voting scale of 7 points or higher. Otherwise, voting was to be repeated after the experts’ discussion and modification of the recommendation.

Two rounds of voting regarding each recommendation were held. The final Consensus recommendations are presented in the text of the article with their justification based on the current evidence before each recommendation.

Full version of the Consensus was presented for the first time in “Pain, Joints, Spine” journal [25] in Ukrainian for wider use by the medical community in Ukraine.

## 3. Consensus Recommendations

### 3.1. Epidemiological Studies in Ukraine on Vitamin D Status in Adults

During 2011–2022, some national and regional epidemiological studies concerning vitamin D status were performed in Ukraine, involving subjects of different ages, sexes, and places of residence [26,27,28,29,30,31]. In the first national study (1575 persons aged 20–89 years) [26], VDD was detected in 81.8%, vitamin D insufficiency (VDI) in 13.6%, and normal 25(OH)D levels in 4.6% of the subjects. The mean serum 25(OH)D level in this study consisted of 35 nmol/L (14 ng/mL). In 2017, the study with a participation of 3460 subjects with musculoskeletal diseases aged 1–92 years [27] demonstrated a higher 25(OH)D blood level (26 ng/mL) compared to the previous study, with a decreased proportion of the VDD (38%) and the VDI (31%).

The third large epidemiological study, performed in 2016–2022 [28] and consisting of the results of 8426 adults aged 20–99 years, showed increased average serum 25(OH)D levels (31 ng/mL) compared to the results of two previous studies. A decreased proportion of VDI (27%) and VDD (20%) was also established. During the COVID-19 pandemic [31] serum 25(OH)D levels in the Ukrainian population were higher (annual index in 2020—36.8 ng/mL, in 2021—35.0 ng/mL) compared to the previously published studies and to the level in 2018 (30.2 ng/mL) [28]. All Ukrainian studies have shown a dependence of 25(OH)D levels on age, with the minimum levels in older age groups, and seasonal fluctuations of 25(OH)D levels with the maximum levels in early fall and late summer and the lowest levels in early spring and late winter.

**Recommendation** **No. 1** [Consensus voting scale (CVS) (agreement level (AL)): 9 (100%)]: *Taking into account the high prevalence of VDD and VDI in the adult population of Ukraine, we recommend increasing awareness in the community about the current situation and positive pleiotropic (skeletal and extra-skeletal) effects of vitamin D in the human body to increase timely screening, adequate prevention, and treatment of VDD*.

### 3.2. Screening of VDD in Adults

The best index for assessing vitamin D status in a human body [32,33] is total 25(OH)D, the main vitamin D circulating form. It has an average half-life of approximately 13–15 days and accounts for the 25(OH)D_2_ and 25(OH)D_3_ levels. For the assessment, blood should be drawn in the morning on an empty stomach. Units of measurement are nmol/L or ng/mL (conversion index is ×2.5). 25(OH)D levels can be measured in various biological fluids (blood, urine, amniotic, and synovial fluids) in experimental conditions as well as in cell cultures, but in clinical practice only serum is used to assess vitamin D status [34]. To measure 25(OH)D levels, various methods based on immunoassays (CLIA, ECLIA, RIA, and ELISA), which are often used in clinical practice due to automation and rapid results, or chromatography (HPLC and LC-MS, which allow the determination of vitamin D metabolites) can be used. However, the last ones are more difficult due to technical equipment, laborious sample preparation, and evaluation. Undoubtedly, standardization of 25(OH)D results and laboratory quality assurance are extremely important [34,35].

It should be noted that various international recommendations [15,18,21,22,36] use different thresholds of VDD definitions. Some studies [37] demonstrated an inverse relationship between the serum parathyroid hormone (PTH) level and 25(OH)D at levels less than 75 nmol/L (<30 ng/mL), which confirms our choice of this value as the threshold for establishing the optimal level. Some guidelines recommend a lower VDD threshold based on the use of meta-regression analysis (MRA), which is less appropriate to note the dose–response relationship based on MRA, instead of individual participant data (IPD) analysis (the last one is recommended by Cochrane experts for developing serum 25(OH)D levels) [38].

The hormonally active vitamin D form (1α,25-dihydroxyvitamin D, 1α,25(OH)_2_D)) has genomic and non-genomic mechanisms, however, its determination is not appropriate for testing vitamin D status and screening for VDD. In subjects with VDD, its level can be normal or even increased, particularly in the case of increased PTH biosynthesis. However, the 1α,25(OH)_2_D serum level is important in subjects with congenital/acquired disorders of phosphate, patients with chronic kidney disease (CKD), vitamin D-resistant rickets and oncogenic osteomalacia, certain types of lymphoma, and chronic granulomatous diseases.

**Recommendation** **No. 2** [CVS (AL): 9 (80%), 7 (13%), 5 (7%)]: *We recommend using a serum level of total 25(OH)D as a laboratory marker for the diagnosis of VDD with the following criteria for determination of vitamin D status:*

- *VDD: <50 nmol/L (<20 ng/mL);*
- *VDI: ≥50 nmol/L (≥20 ng/mL) and <75 nmol/L (<30 ng/mL);*
- *Sufficient level of vitamin D: 75–125 nmol/L (30–50 ng/mL);*
- *Safe but not target level of vitamin D: >125–150 nmol/L (>50–60 ng/mL);*
- *Zone of uncertainty with potential benefits or risks for vitamin D: >150–250 nmol/L (>60–100 ng/mL);*
- *Excess/toxicity zone of vitamin D: >250 nmol/L (>100 ng/mL).*

The wider recognition of the positive effects of vitamin D in the human body has led to an increased number of 25(OH)D tests in recent years worldwide [39,40]. However, there is still insufficient evidence for its use to support the feasibility of universal screening for VDD [41,42,43]. Nevertheless, there is strong evidence indicating an elevated prevalence of VDD among older age subjects [27,28], persons with darker skin pigmentation [44], obesity, and metabolic syndrome [27,45]. High VDD was demonstrated in patients with metabolic bone disorders [46], autoimmune diseases [47,48,49], including rheumatic disorders [50,51,52,53], inflammatory bowel diseases [54,55], infectious diseases [56,57], endocrine system disorders (type I diabetes mellitus, thyroid diseases, etc.) [58,59,60], cardiovascular [61,62,63], nervous system disorders [64,65,66], kidney diseases [67], chronic obstructive pulmonary disease [68], and tumors [69]. Also, for patients who take some drugs on a long-term basis, a negative impact on vitamin D metabolism was observed [70,71]. Therefore, assessing the serum 25(OH)D levels in this group of subjects can prove to be valuable for the timely assessment of VDD, its effective prevention, and treatment.

**Recommendation** **No. 3** [CVS (AL): 9 (80%), 7 (20%)]: *We do not recommend assessment of 25(OH)D serum level in adults without apparent indications, but we recommend it in persons from the following risk groups:*

- *Persons with dark skin pigmentation;*
- *Obese subjects (body mass index ≥ 30 kg/m^2^);*
- *Pregnant and lactating females;*
- *Older subjects (≥60 years old);*
- *Subjects with bone or muscle pain;*
- *Older subjects with a high risk of falls and a history of low trauma fractures;*
- *Patients with metabolic bone diseases (osteoporosis and osteomalacia);*
- *Immobilized persons and subjects during prolonged hospitalization;*
- *Patients with liver or kidney failure;*
- *Patients with endocrine disorders (I and II types of diabetes mellitus; hyperparathyroidism; thyroid diseases, etc.);*
- *Subjects with malabsorption syndromes (inflammatory bowel diseases, cystic fibrosis, enteritis after radiation, conditions after bariatric surgery, etc.);*
- *Patients with chronic autoimmune diseases (rheumatoid arthritis, systemic lupus erythematosus, multiple sclerosis, etc.);*
- *Patients with malignancy;*
- *Subjects with granulomatous diseases (sarcoidosis, tuberculosis, histoplasmosis, berylliosis, coccidioidomycosis, etc.);*
- *Persons with prolonged use of drugs with a negative impact on vitamin D metabolism (glucocorticoids, anticonvulsants, hypocholesterolemic, antifungal medications, AIDS drugs, etc.).*

Nowadays, it is well known that vitamin D plays an important role in calcium–phosphorus homeostasis and bone metabolism [5,12,72]. In chronic VDD, intestinal absorption of calcium, phosphate, and magnesium decreases [73], and calcium and phosphate ion reabsorption in the renal tubules is impaired. In an intestine, 1α,25(OH)_2_D induces calcium absorption through the TRPV6 (transient potential of vanilloid type 6) channel, and in a kidney, it mediates active calcium reabsorption through TRP5 by a mechanism similar to intestinal calcium absorption, while the transcription of the gene encoding TRPV6 is regulated through the vitamin D/VDR/RXR (retinoid X receptor) pathway [74].

Elevated PTH levels in the setting of VDD lead to bone resorption. This process is accompanied by the release of calcium and phosphate into the bloodstream and the reabsorption of calcium in the kidneys to uphold its serum blood level. It should be noted that VDD is usually accompanied by normal serum calcium and phosphorus levels. PTH and total alkaline phosphatase (ALP) levels may be either in the upper normal range or increased, which may be accompanied by a low calcium excretion rate in the daily urine. However, with high ALP levels, the development of secondary hyperparathyroidism, hypocalcemia, and/or hypophosphatemia may be present in subjects with severe or prolonged VDD [46,72]. Elevated ALP serum blood levels can be considered a marker of osteomalacia; in VDD, ALP is produced by osteoblasts within a broad layer of osteoid that develops under conditions of compromised mineralization [75].

Low serum blood magnesium (Mg) levels have a significant effect on vitamin D metabolism [76]. Additionally, Mg deficiency is a key factor in the occurrence of Mg-dependent rickets or osteomalacia. Some forms of this pathology are resistant to vitamin D, and the prescription of Mg supplements significantly increases the efficacy of VDD therapy [77].

For a comprehensive assessment of vitamin D status in people, serum creatinine levels should be also determined. This important index reflects the kidney functional state, where the second stage of vitamin D synthesis occurs with the formation of its hormonally active form (1α,25(OH)_2_D). Increased serum blood creatinine levels may indicate acute or chronic renal failure and other kidney diseases. In addition, severe VDD acts as a risk factor for decreased calcitriol production in the proximal tubules of kidneys, which increases the risk of subsequent renal disorders, such as nephrosclerosis [78].

**Recommendation** **No. 4** [CVS (AL): 9 (80%), 7 (20%)]: *In subjects with VDD, we recommend interpretation of 25(OH)D levels together with serum level of calcium, phosphorus, parathyroid hormone, alkaline phosphatase, magnesium, and creatinine.*

### 3.3. Prevention and Treatment of VDD in Adults

Taking into account that vitamin D is ingested through food, synthesized in the skin, and can be supplied in various vitamin D supplements, balanced nutrition, healthy lifestyle, outdoor activities, and appropriate levels of physical activity are key strategies for VDD prevention. Current evidence confirms that the expediency of prescribing vitamin D prophylactic doses in adults after measuring its serum level should be determined by the season of the year, taking into account age, body mass index, dietary preferences, physical activity of the subjects, as well as the presence of other risk factors for VDD.

Vitamin D_2_ and vitamin D_3_ are most commonly used to prevent and treat VDD and VDI [79]. Vitamin D_3_ is characterized by a low affinity for plasma vitamin D-binding protein. Additionally, vitamin D_3_ has a higher rate of 25-hydroxylation in the liver and subsequent hydroxylation to form active metabolites in kidneys, higher discrimination coefficient (predominance of activity) [2,4,5], and higher efficacy, which is confirmed by modern randomized clinical trials and meta-analyses [80,81].

Current evidence confirmed that vitamin D should be prescribed with adherence and taking into account particularities of the subject’s diet, functional status, gastrointestinal pathology, etc. Nowadays, different regimens of vitamin D supplementation are used worldwide (monthly, quarterly, semi-annually, and annually). According to many researchers [15,82,83], daily and weekly regimens of vitamin D intake are more suited than bolus ones. In Ukraine, vitamin D is available only in various oral forms (capsules, drops, and tablets) and can be administered through daily or weekly regimes.

**Recommendation** **No. 5** [CVS (AL): 9 (67.7%), 7 (33.3%)]: *For the prevention and treatment of VDD we recommend the prescription of the oral form (daily or weekly) of cholecalciferol (vitamin D_3_); alternatively (vegetarianism, veganism, etc.), we recommend the oral form of ergocalciferol (vitamin D_2_).*

For persons aged 19–65 years without risk factors that affect vitamin D metabolism, most international guidelines dedicated to the management of VDD [15,18,21,22,36] recommend, if possible, to receive vitamin D from May to September through sun exposure for at least 15 min from 10.00 to 15.00 and diets that include vitamin D rich foods. Vitamin D supplementation using various doses depending on the body mass, season, and food preferences may be recommended if the above is limited or impossible. Epidemiological studies conducted in Ukraine on seasonal fluctuations in 25(OH)D levels [25,27,28,31] have shown that lower vitamin D levels were observed in late autumn, winter, and spring. To reduce the VDD, it may be important to prescribe prophylactic doses from October to April.

In accordance with the current international guidelines [15,18,21,22,36], there is no clear Consensus concerning the prescription of prophylactic doses of prescription of cholecalciferol (200 to 2000 IU/d). Pharmacodynamic studies have shown that taking 100 IU/d of cholecalciferol leads to an increase in serum 25(OH)D levels by an average of ~1 ng/mL (2.5 nmol/L) [84]. However, this index can be significantly affected by various external and internal factors. Vitamin D supplementation is often prescribed according to age. According to the EFSA [85], the recommended cholecalciferol intake consists of 600 IU/d with an upper intake limit (for children aged 1 year and over 11 years and adults) of 4000 IU.

The existing data devoted to differences in vitamin D doses to achieve a 25(OH)D level of 50 nmol/L in ≥97.5% of subjects [86], depending on the approach used in analysis (according to the MRA, it was 560 IU/d, and the IDU, respectively, 1040 IU/d), allowed us to recommend a dose of at least 800 IU/d as the target for the prevention of VDD.

**Recommendation** **No. 6** [CVS (AL): 9 (66.7%), 7 (26.7%), 5 (6.6%)]: *For healthy adults without diseases and conditions affecting vitamin D metabolism in the human body, we recommend the prescription of cholecalciferol at a dose of 800–2000 IU/d (depending on body weight) from October to April due to a decreased level of endogenous vitamin D synthesis in the skin during this period.*

As was noted above, many but not all papers confirmed that the frequency of VDD increases in older persons. This is due to decreased vitamin D synthesis in the skin during aging (thickening of the stratum corneum, decreased vitamin D receptor density, etc.), and impaired vitamin D absorption from food. It can have a negative effect on the risk of skeletal and extra-skeletal vitamin D-related disorders. Moreover, immobilization leads to the development of VDD, especially during prolonged hospitalization with limited functional activity. Current recommendations for vitamin D intake in older adults vary, but most of them, including the Order of the Ministry of Health of Ukraine [87], note the need to increase daily vitamin D supplementation.

**Recommendation** **No. 7** [CVS (AL): 9 (87%), 7 (13%)]: *For the elderly, immobilized persons, and subjects with prolonged hospitalization with limited functional activity, we recommend the prescription of cholecalciferol in the dose of 800–2000 IU/d during the year.*

Current international guidelines [15,18,21,22,36] recommend a wide range (200–2000 IU/d) of cholecalciferol in women during pregnancy and lactation. Although, according to the Order of the Ministry of Health of Ukraine [87], increased vitamin D supplementation during pregnancy is not recommended. Nevertheless, we consider it advisable to increase vitamin D supplementation for females during pregnancy and lactation given the increased need for women. Optionally, before prescription, serum 25(OH)D levels should be checked to choose the optimal cholecalciferol dose. If testing is not possible, depending on diet, lifestyle, diseases, and conditions affecting vitamin D metabolism, additional cholecalciferol supplementation at a dose of 800–2000 IU/d may be considered.

**Recommendation** **No. 8** [CVS (AL): 9 (87%), 7 (13%)]: *For females planning a pregnancy we recommend the prescription of cholecalciferol at a dose of 800–2000 IU/d and continue cholecalciferol use throughout pregnancy and lactation.*

Prophylactic administration of vitamin D (800–2000 IU/day) might be insufficient to sustain optimal serum 25(OH)D concentrations in individuals with obesity (body mass index > 30 kg/m^2^), dark skin color, and those with diseases or conditions linked to impaired vitamin D metabolism. Therefore, these subjects need higher cholecalciferol doses (3000–5000 IU/d) depending on dietary preferences, body weight, and season, with the need for individual monitoring of serum 25(OH)D levels. Additionally, the results of numerous studies and meta-analyses demonstrated a link between vitamin D supplementation and reduced risk of various diseases and conditions (infectious [88], chronic autoimmune [89], diabetes [90,91], cancer [92,93]), as well as mortality [69]).

**Recommendation** **No. 9** [CVS (AL): 9 (80%), 7 (13%), 5 (7%)]: *In the patients with diseases or conditions affecting vitamin D metabolism in the human body, we recommend the individual selection of a cholecalciferol prophylactic dose (3000–5000 IU/d) with achievement and maintenance of optimal 25(OH)D serum levels.*

Current data suggest that it is possible to use higher doses (up to 10,000 IU/d) of the cholecalciferol if necessary to quickly correct the VDD in the case of osteomalacia, severe vitamin VDD (<10 ng/mL), etc. Also, it is possible, if necessary, in patients with increased fracture risk, secondary hyperparathyroidism [15,21], and the necessity for prescribing antiosteoporotic therapy. This approach is effective and safe [94]. However, tailoring of high daily vitamin D doses should be performed individually depending on the season, the person’s functional activity regimen, and the presence of comorbidities or conditions that affect vitamin D metabolism.

**Recommendation** **No. 10** [CVS (AL): 9 (66.7%), 7 (26.7%), 5 (6.6%)]: *For subjects with confirmed VDD and without diseases or conditions affecting vitamin D metabolism, we recommend starting the treatment with higher doses (4000–7000 IU/d) of cholecalciferol compared to the prophylactic doses recommended for the overall population.*

**Recommendation** **No. 11** [CVS (AL): 9 (66.7%), 7 (26.7%), 5 (6.6%)]: *For the subjects with confirmed VDD and diseases and conditions that affect vitamin D metabolism, we recommend starting the treatment using higher cholecalciferol doses (up to 10,000 IU/d) compared to doses recommended for healthy adults without additional risk factors.*

According to the current international guidelines [15,18,21,22,36], a 25(OH)D level of less than 50 nmol/L (<20 ng/mL) is the threshold for VDD, which determines the need for treatment. However, it is important to acknowledge that in the case of a lower initial 25(OH)D level, its rate of increase tends to be faster, and the curve of concentration growth becomes less pronounced when it approaches its optimal values.

Nowadays, there is no full agreement concerning the timing of monitoring of serum 25(OH)D levels. According to some studies, serum 25(OH)D levels should be monitored 4–12 weeks after the initiation of VDD treatment [33,94,95], but other authors proposed a much longer period (3–6 months) [96]. The VDD severity and the presence of diseases or conditions that affect vitamin D metabolism should be taken into account when planning monitoring, the timing of which may differ depending on the form and regimen of vitamin D administration.

**Recommendation** **No. 12** [CVS (AL): 9 (73%), 7 (20%), 5 (7%)]: *We recommend initiating the treatment of VDD at the serum 25(OH)D levels less than 50 nmol/L (<20 ng/mL). The treatment duration should be 4–12 weeks depending on the severity of VDD and other risk factors and should be continued until the target serum 25(OH)D level (75–125 nmol/L or 30–50 ng/mL) is reached. Afterward, we recommend using lower doses of cholecalciferol (800–2000 IU/d) to maintain optimal vitamin D status.*

The decision concerning additional vitamin D supplementation (in the case of VDI (25(OH)D < 75 nmol/L (or <30 ng/mL)) should be performed individually. The need for rapid correction of VDD and other indications should be taken into account.

It is important to consider the use of not only cholecalciferol (vitamin D_3_) and ergocalciferol (vitamin D_2_), which are the most common, but also some other vitamin D metabolites for the prevention and treatment of VDD [97]. However, it should be borne in mind that calcitriol (1α,25(OH)_2_D) and its analogs (α-calcidol) are associated with an increased risk of hypercalcemia, their prescription should be important in patients with bone and mineral disorders associated with CKD or chronic hyperparathyroidism [98].

**Recommendation** **No. 13** [CVS (AL): 9 (60%), 7 (27%), 5 (13%)]: *We do not recommend active vitamin D metabolites for the treatment of VDD in subjects without diseases and conditions that affect vitamin D metabolism, however, we recommend them for the treatment of VDD in patients with chronic hyperparathyroidism or bone and mineral disorders associated with CKD.*

### 3.4. Vitamin D in the Treatment of Musculoskeletal Diseases

Osteoporosis is a common disease associated with an increased risk of fragility fractures. The combined use of cholecalciferol and calcium has been shown to increase bone mineral density and reduce the risk of osteoporotic fractures as well as osteomalacia [79,99,100]. The use of calcium and cholecalciferol in the treatment of osteoporosis is supported by extensive scientific evidence based on randomized clinical trials and meta-analyses, which have shown a statistically and clinically significant reduction in the risk of osteoporotic fractures [101,102,103]. Current international guidelines recommend using the combined supplementation with cholecalciferol (400–800 IU/d) and calcium with antiosteoporotic drugs in the treatment of postmenopausal osteoporosis in women [104,105], men [106], and glucocorticoid-induced osteoporosis [107].

The positive effect of daily cholecalciferol use (800–1000 IU/d) on the reduction of fracture and fall risk was noted, while intermittent supplementation was not effective [108].

According to the recommendations of international scientific societies, the use of calcium and D supplements in the therapeutic treatment of osteoporosis in the following doses is effective: European Society for Clinical and Economic Aspects of Osteoporosis (ESCEO) in conjunction with the Advisory Committees and National Societies of the International Osteoporosis Foundation (IOF) (2019, 800 IU); National Osteoporosis Guideline Group (NOGG) (2017), 800 IU); National Osteoporosis Foundation (NOF) (800–1000 IU), and the joint American Association of Clinical Endocrinologists/American College of Endocrinology (AACE/ACE) (2016, 1000–2000 IU) [109]. However, according to the recent systematic review, it was noted that in postmenopausal women at increased risk of osteoporosis and VDD, only daily doses of 2000 IU/day of vitamin D consistently increased 25(OH)D levels above 30 ng/mL [110].

The use of vitamin D in combination with calcium increases the effectiveness of antiosteoporotic therapy, in particular when using bisphosphonates [111] or denosumab [112,113]. In addition, to improve the safety profile of acute-phase reactions [114,115] caused by these drugs, the use of this complex is also recommended. Given the essential role of vitamin D, it is important to determine the serum level of 25(OH)D before initiating antiosteoporotic therapy in patients with osteoporosis.

**Recommendation** **No. 14** [CVS (AL): 9 (66.7%), 7 (26.7%), 5 (6.6%)]: *For patients with osteoporosis and its complications we recommend determining the serum 25(OH)D level before initiating antiosteoporotic therapy to improve its effectiveness and safety profile.*

If the VDD is detected before antiosteoporotic therapy, we recommend its correction before starting antiosteoporotic treatment.

If the serum 25(OH)D level is normal, we recommend the prescription of cholecalciferol (800–2000 IU/d) in combination with calcium (1000 mg/d of elemental calcium) throughout the course of antiosteoporotic therapy.

For patients with an increased risk of falls or fractures (according to the Ukrainian version of FRAX) we recommend the prescription of cholecalciferol (800–2000 IU/d) throughout the year.

## 4. Conclusions

Despite a recently decreased frequency of VDD in the Ukrainian population, the expert group recommends raising awareness among the medical and public community regarding VDD’s importance and improving the strategies for its decline. We recommend screening the serum total 25(OH)D level in separate risk groups while maintaining a goal concentration of 75–125 nmol/L (30–50 ng/mL). Also, we recommend prophylactic cholecalciferol prescription (800–2000 IU/d for young, healthy subjects, and 3000–5000 IU/d for persons with diseases or conditions with negative effects on vitamin D metabolism). For the VDD treatment, the expert group recommends the short-term administration of higher cholecalciferol doses (4000–10,000 IU/d) with monitoring of 25(OH)D levels after 4–12 weeks of treatment, followed by the use of maintenance doses. Furthermore, we recommend the assessment of serum 25(OH)D levels before initiating therapy in patients with osteoporosis and providing a prescription of cholecalciferol (800–2000 IU/d) in combination with calcium (1000 mg/d of elemental calcium) throughout the full course of antiosteoporotic treatment (Table A1).

## Data Availability

Not applicable.

## Appendix A

**Table A1:** Recommended vitamin D doses for various groups of adults.

Target Population	Recommended Vitamin D Doses
Healthy adults without diseases and conditions affecting vitamin D metabolism	800–2000 IU/d (depending on body weight) from October to April
Elderly, immobilized persons and subjects with prolonged hospitalization with limited functional activity, increased risk of falls or fractures (according to the FRAX questionnaire)	800–2000 IU/d during the year
Females planning a pregnancy and during the pregnancy and lactation	800–2000 IU/d during the pregnancy and lactation
Patients with diseases or conditions affecting vitamin D metabolism	3000–5000 IU/d with individual selection of the dose and duration of supplementation
Subjects with confirmed VDD and without diseases or conditions affecting vitamin D metabolism	4000–7000 IU/d with personal selection of the dose and duration of supplementation
Subjects with confirmed VDD and diseases and conditions that affect vitamin D metabolism	Up to 10,000 IU/d with individual selection of the dose and duration of supplementation
Patients with osteoporosis and its complications with normal serum 25(OH)D level	800–2000 IU/d in combination with calcium (1000 mg/d of elemental calcium) throughout antiosteoporotic treatment

Notes. VDD—Vitamin D deficiency; 25(OH)D—25-hydroxyvitamin D.
